# Comparison of different estimated glomerular filtration rates for monitoring of kidney function in oncology patients

**DOI:** 10.1093/ckj/sfae006

**Published:** 2024-01-12

**Authors:** Tijl Vermassen, Karen Geboes, Nicolaas Lumen, Charles Van Praet, Sylvie Rottey, Joris Delanghe

**Affiliations:** Department of Medical Oncology, University Hospital Ghent, Ghent, Belgium; Biomarkers in Cancer, Ghent University, Ghent, Belgium; Cancer Research Institute Ghent, Ghent, Belgium; Cancer Research Institute Ghent, Ghent, Belgium; Digestive Oncology, Department of Gastroenterology, Ghent University Hospital, Ghent, Belgium; Department of Internal Medicine and Pediatrics, Ghent University, Ghent, Belgium; Cancer Research Institute Ghent, Ghent, Belgium; Department of Urology, University Hospital Ghent, Ghent, Belgium; Department of Human Structure and Repair, Ghent University, Ghent, Belgium; Cancer Research Institute Ghent, Ghent, Belgium; Department of Urology, University Hospital Ghent, Ghent, Belgium; Department of Human Structure and Repair, Ghent University, Ghent, Belgium; Department of Medical Oncology, University Hospital Ghent, Ghent, Belgium; Biomarkers in Cancer, Ghent University, Ghent, Belgium; Cancer Research Institute Ghent, Ghent, Belgium; Drug Research Unit Ghent, University Hospital Ghent, Ghent, Belgium; Cancer Research Institute Ghent, Ghent, Belgium; Department of Diagnostic Sciences, Ghent University, Ghent, Belgium

**Keywords:** cystatin C, glomerular filtration rate estimation, kidney function, renal cell carcinoma, tyrosine kinase inhibitors

## Abstract

**Background:**

Tyrosine kinase inhibitors (TKIs) are associated with kidney function deterioration. A shift is ongoing towards glomerular filtration rate (GFR) equations based on other protein markers, such as cystatin C (CSTC) and β-trace protein (BTP). We evaluated various GFR equations for monitoring of kidney function in actively treated oncology patients.

**Methods:**

We monitored 110 patients receiving a TKI. Blood and urine were collected during therapy. Serum analysis included creatinine (Cr), CSTC and BTP; for consequent GFR determination. Urine was analysed for protein, albumin, immunoglobulin G, and α-1-microglobulin. A similar analysis was done in a patient subgroup receiving immune checkpoint inhibitors (ICI) as prior or subsequent line of therapy.

**Results:**

Cr remained constant during TKI treatment (*P *= 0.7753), whereas a significant decrease in CSTC (from week 2 onward, *P *< 0.0001) and BTP (at weeks 2 and 4, *P *= 0.0100) were noticed. Consequently, GFR estimations, using CSTC and/or BTP as a biochemical parameter, showed an apparent increase in GFR, whereas this was not observed for Cr-related GFR estimations. As a result, the GFR gap (ΔGFR) was significantly different from week 2 onward between Cr-based and CSTC-based GFR and between BTP-based and CSTC-based GFR. Glomerular damage was noticed with significant increase in urine protein-to-creatinine ratio, albumin-to-creatinine ratio and immunoglobulin G (all *P *< 0.0001). No change in α-1-microglobulin was seen. ICI treatment had no effect on Cr (*P *= 0.2262), CSTC (*P *= 0.7341), and BTP concentrations (*P *= 0.3592).

**Conclusion:**

GFR equations, in which CSTC is incorporated, fail to correctly estimate the GFR in oncology patients treated with TKIs. As TKI-treated patients show clear signs of glomerular injury, further assessment is needed on how to correctly monitor the kidney function in actively treated oncology patients.

## Graphical Abstract

**Graphical Abstract:**
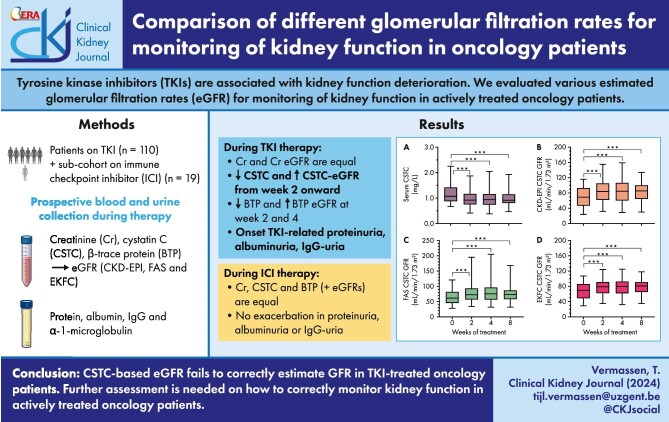

KEY LEARNING POINTS **What was known**:

- Tyrosine kinase inhibitors (TKI) induce kidney damage.
- No data exists on the most appropriate formula for estimated glomerular filtration rate (eGFR) in TKI-treated oncology patients.
- Comparison between eGFR equations was made to optimize kidney function monitoring in oncology patients treated with targeted agents.

**This study adds**:

- Creatinine-based eGFR remains constant in treatment with TKIs.
- Cystatin C (CSTC) decreases after TKI administration, probably due to a tumor-related process (release of cysteine proteinase).
- CSTC-based eGFR, irrespective of the formula used, overestimated kidney function of oncology patients treated with TKIs.

**Potential impact**:

- The eGFR cannot be trusted blindly, especially the CSTC-based eGFR, during follow-up of oncology patients under TKI treatment.
- Guidelines should discommend the use of CSTC-based eGFR as a kidney monitoring tool in oncology patients treated with a TKI.

## INTRODUCTION

Tyrosine kinase inhibitors (TKIs) are frequently used in the metastatic setting of various solid carcinoma. TKIs can be subdivided into several classes depending on their target, although most TKIs have multiple targets [1–5]. Due to their broad spectrum of activity and the presence of these targets in normal renal tissue, the onset of TKI-related kidney injury has been reported in several trials [6–9]. Therefore, it is essential to correctly monitor the kidney function in oncology patients treated with TKIs.

The most commonly used analyte to measure kidney function is creatinine (Cr). Cr (113 Da) is the metabolite of muscle creatine phosphate. It is produced at a fairly constant rate, and entirely excreted via the kidney by being freely filtered across the glomerular membrane with no tubular reabsorption. Serum Cr measurement has been historically used and recommended as a marker for renal function through glomerular filtration rate (GFR) estimation [10–13]. However, there have recently been drastic changes in the diagnostic landscape for kidney function assessment. Several formulas have been established to determine the Cr-based estimated GFR (eGFR), some of these excluding race and other potentially confounding demographic factors from the equation [14–18]. Moreover, a shift is ongoing in the last decade towards the more extensive use of other serum analytes in GFR equations [19–23]. Cystatin C (CSTC), a low-molecular-weight protein of 13.3 kDa, is regarded as a good filtration marker due to its physical and chemical properties, its constant rate of production, its relatively freely filtering by the glomeruli, and its reabsorption by and catabolism in the proximal renal tubular cells. Consequently, formulas have been developed, incorporating CSTC next to Cr, for the estimation of the GFR, which are considered to be more robust, as they are not influenced by several confounding factors, such as race and body mass [15, 16, 18, 22, 24]. Another endogenous marker for kidney function evaluation is lipocalin prostaglandin D2 synthase or β-trace protein (BTP). BTP (23–29 kDa) is generated at a constant rate by glial cells in the brain and is freely filtered through the glomerulus with minimal non-renal elimination. Several equations have been elaborated to calculate GFR based on BTP measurement [15, 16, 25–27].

As a correct and close monitoring of kidney function is crucial in TKI-treated oncology patients, it is imperative to determine which GFR equations are most optimal during patient follow-up. Therefore, we evaluated and compared several GFR equations for use as a kidney function monitoring tool in actively treated oncology patients.

## MATERIALS AND METHODS

### Subjects and samples

A cohort of 110 patients was prospectively recruited and monitored at the University Hospital Ghent’s Departments of Medical Oncology and Gastroenterology. All patients received a TKI as active treatment regimen (sunitinib, pazopanib, sorafenib, axitinib, or cabozantinib). Additionally, a subcohort was created of 19 patients who received an ICI in monotherapy (nivolumab) prior to or after their TKI regimen to determine if similar outcomes can be seen for TKI versus ICI. The STARD diagram is given in Fig. S1 (see online supplementary material). Informed consent was given by all participants and the study was approved by the local Ethics Committee (Belgian registration: B670201214356). Upon start of treatment, clinical and biochemical evaluations were performed at baseline, after 2, 4, 8, 12, and 24 weeks of therapy. One serum tube (5 mL) and a spot urine sample was collected at each visit. Serum (following coagulation) and urine were centrifuged at 2000 *g* for 10 minutes and used for biochemical analysis.

### Methods

We used ARCHITECT c16000 clinical chemistry analyser (Abbott, Chicago, IL, USA) to measure serum Cr (IDMS-based kinetic alkaline picrate methodology, calibrated against the international SRM-967 standard), total urinary protein, Cr, and α-1-microglobulin. A particle-enhanced nephelometric assay (PENIA) on a BN Nephelometer II analyser (Siemens Healthcare, Marburg, Germany) was used to determine serum CSTC (calibrated against the certified standard ERM®-DA471/IFCC), serum BTP [calibrated according to kit internal protein reference preparation (N Latex BTP, 11528254_en Rev. 07–Outside USA, Siemens Healthcare Diagnostics Products GmbH)], and albumin and immunoglobulin G (IgG) in urine. All reagents and controls are commercially available. The protein and albumin concentrations were expressed as urine protein-to-creatinine ratio (UPCR) and urine albumin-to-creatinine ratio (UACR; mg/g urinary Cr).

GFR was estimated using various equations. Cr-based GFR was calculated using the CKD-EPI, FAS, and EKFC equation [14–18, 28]. Based on the recommendations for European nephrology, only the CKD-EPI 2009 equation was used [29]. CSTC-based GFR was calculated via the CKD-EPI, FAS, and EKFC formula [15, 16, 18, 24]. In addition, the combined GFR for Cr and CSTC was determined using the CKD-EPI 2012, and FAS equation [14–16]. Lastly, the BTP-associated GFR was calculated by means of the CKD-EPI and FAS formula, as well as the combinations with Cr and/or CSTC using the FAS formulas [15, 16, 25]. All formulas used in this paper have been summarized in Table S1 (see online supplementary material). The difference in GFR (ΔGFR) estimations, using only one biochemical analyte, was determined by subtracting the CSTC-based eGFR from the Cr-based eGFR, the BTP-based eGFR from the CSTC-based eGFR, and the BTP-based eGFR from the Cr-based eGFR, respectively.

### Statistical analysis

Considering statistical significance, only cases without missing values were selected, leading to data cut-off taken after 8 weeks of therapy with 74 patients included in the statistical analysis. Intrapatient differences during treatment (baseline versus week 2 versus week 4 versus week 8) were determined using the non-parametric Friedman test. Intergroups differences were further analysed using Dunn's testing. Next, linear mixed models for variance and covariance were established for all patients. In addition, for each GFR estimation, a regression model was visualized based on the median eGFR at each time point to illustrate the course of eGFR during treatment follow-up. *P*-values <0.05 were considered statistically significant. Statistical analyses were performed with MedCalc v20.110 (MedCalc Software, Ostend, Belgium), SPSS Statistics v29.0.1.0 (IBM Corp., Armonk, NY, USA), and GraphPad Prism v8.0.2 (GraphPad Software Inc., La Jolla, CA, USA).

## RESULTS

### Patients’ characteristics

Patients’ characteristics are summarized in Table 1. The majority of patients were male (59%) and suffered from mRCC (85%). Therapeutic options consisted of sunitinib (32%), pazopanib (20%), cabozantinib (19%), axitinib (15%), and sorafenib (14%). Due to TKI administration, median systolic blood pressure in treated patients increased from baseline 134 mm Hg to 142 mm Hg, 140 mm Hg, and 139 mm Hg at week 2, 4, and 8, respectively (*P *= 0.0046). The diastolic blood pressure rose from baseline 80 mm Hg to 85 mm Hg at week 2 and 82 mm Hg at week 4, before stabilizing at week 8 (81 mm Hg; *P *= 0.0161).

**Table 1:** Patients’ characteristics.

Parameter	ITT cohort	Statistical analysis cohort
Total	110 (100)	74 (100)
Gender		
Female	43 (39)	30 (41)
Male	67 (61)	44 (59)
Age (years)	64 (36, 83)	65 (36, 83)
Malignancy		
Renal cell carcinoma	95 (86)	63 (85)
Adenoid cystic carcinoma	13 (12)	9 (12)
Pancreatic neuroendocrine tumor	1 (1)	1 (1)
Thyroid carcinoma	1 (1)	1 (1)
TKI administered		
Sunitinib	35 (32)	24 (32)
Pazopanib	25 (23)	15 (20)
Cabozantinib	17 (15)	14 (19)
Axitinib	18 (16)	11 (15)
Sorafenib	15 (14)	10 (14)

All data are given as number (percentage) except for Age: median (range). ITT, intention to treat; TKI, tyrosine kinase inhibitor.

**Figure 1:**
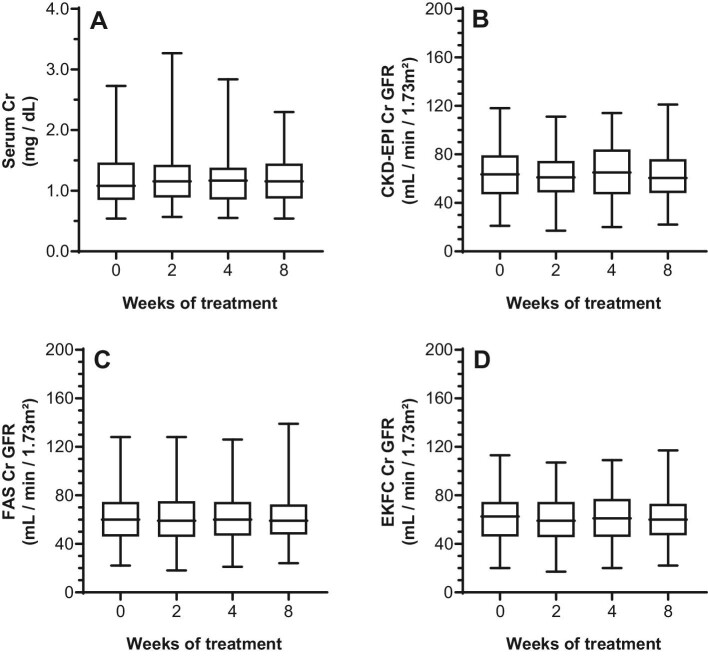
Intrapatient comparison of serum Cr and associated eGFR during TKI treatment. Box plots are given for the different parameters over time. X-axis depicts time of measurement. Comparisons are illustrated for: **A** Cr concentrations (*P *= 0.7753); **B** CKD-EPI Cr-based eGFR (*P *= 0.7457); **C** FAS Cr-based eGFR (*P *= 0.7937); and **D** EKFC Cr-based eGFR (*P *= 0.7453). Cr-based eGFR was estimated using the CKD-EPI 2009 equation. CKD-EPI, Chronic Kidney Disease Epidemiology Collaboration; Cr, creatinine; eGFR, estimated glomerular filtration rate; EKFC, European Kidney Function Consortium; FAS, Full Age Spectrum; TKI, tyrosine kinase inhibitor.

**Figure 2:**
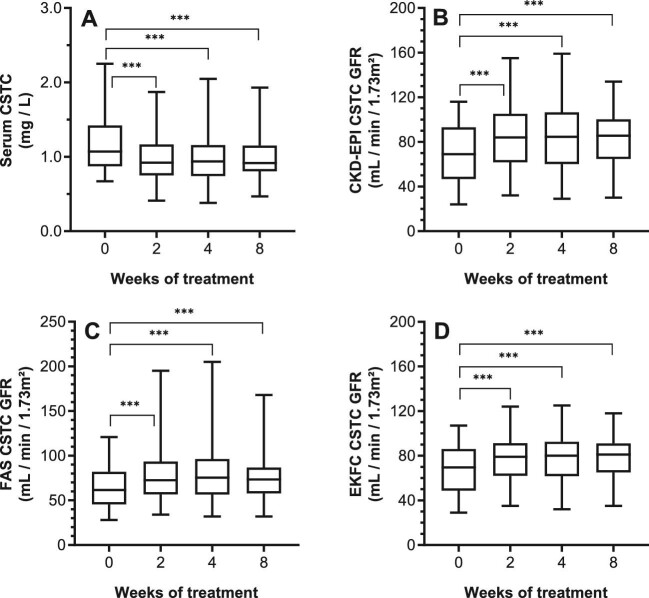
Intrapatient comparison of serum CSTC and associated eGFR during treatment. Box plots are given for the different parameters over time. X-axis depicts time of measurement. Comparisons are illustrated for: **A** CSTC concentrations (*P *< 0.0001); **B** CKD-EPI CSTC-based eGFR (*P *< 0.0001); **C** FAS CSTC-based eGFR (*P *< 0.0001); and **D** EKFC CSTC-based eGFR (*P *< 0.0001). Intergroup significant changes are indicated in the figure (***P < 0.001). CKD-EPI, Chronic Kidney Disease Epidemiology Collaboration; CSTC, cystatin C; eGFR, estimated glomerular filtration rate; EKFC, European Kidney Function Consortium; FAS, Full Age Spectrum; TKI, tyrosine kinase inhibitor.

**Figure 3:**
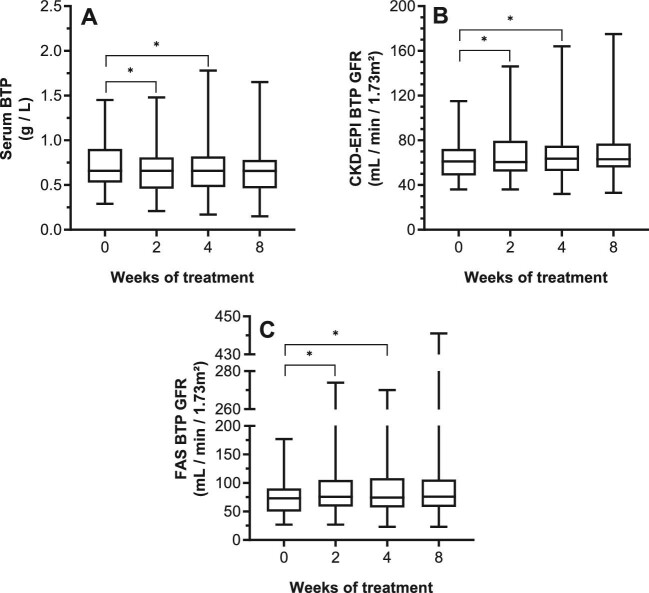
Intrapatient comparison of serum BTP and associated eGFR during treatment. Box plots are given for the different parameters over time. X-axis depicts time of measurement. Comparisons are illustrated for: **A** BTP concentrations (*P *= 0.0100); **B** CKD-EPI BTP-based eGFR (*P *= 0.0089); and **C** FAS BTP-based eGFR (*P *= 0.0070). Intergroup significant changes are indicated in the figure (*P < 0.05). BTP, β-trace protein; CKD-EPI, Chronic Kidney Disease Epidemiology Collaboration; eGFR, estimated glomerular filtration rate; EKFC, European Kidney Function Consortium; FAS, Full Age Spectrum; TKI, tyrosine kinase inhibitor.

**Figure 4:**
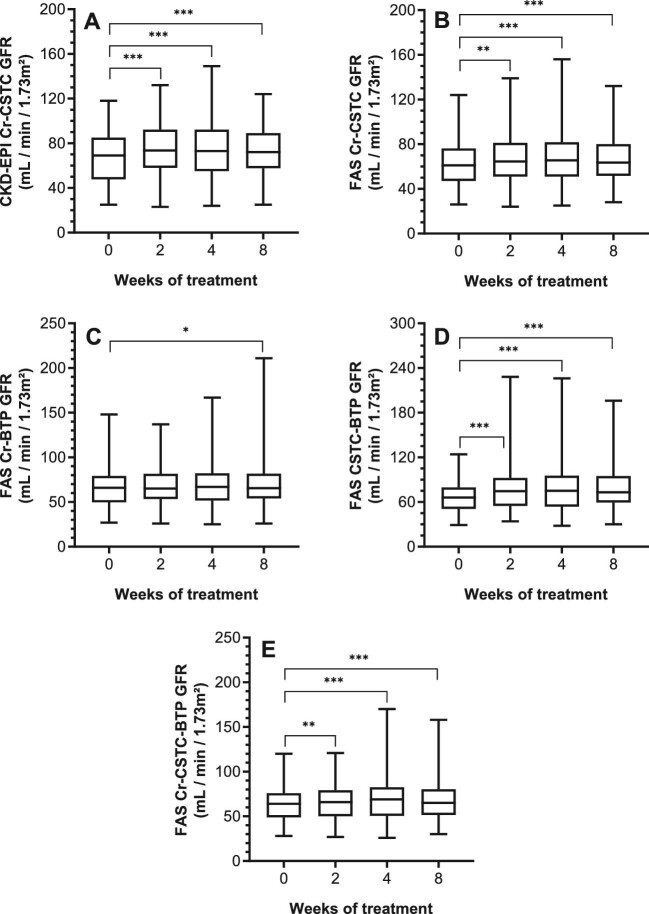
Intrapatient comparison of combined eGFR during TKI treatment. Box plots are given for the different parameters over time. X-axis depicts time of measurement. Comparisons are illustrated for: **A** CKD-EPI Cr-CSTC-based eGFR concentrations (*P *< 0.0001); **B** FAS Cr-CSTC-based eGFR (*P *< 0.0001); **C** FAS Cr-BTP-based eGFR (*P *= 0.0302); **D** FAS CSTC-BTP-based eGFR (*P *< 0.0001); and **E** FAS CSTC-BTP-BTP-based eGFR (*P *< 0.0001). Intergroup significant changes are indicated in the figure (*P < 0.05; **P < 0.01; ***P < 0.001). Cr-CSTC based eGFR was estimated using the CKD-EPI 2012 equation. BTP, β-trace protein; CKD-EPI, Chronic Kidney Disease Epidemiology Collaboration; Cr, creatinine; CSTC, cystatin C; eGFR, estimated glomerular filtration rate; EKFC, European Kidney Function Consortium; FAS, Full Age Spectrum; TKI, tyrosine kinase inhibitor.

Baseline serum analyte concentrations and respective eGFRs, and changes through therapy are given in Table 2. Serum Cr and associated eGFRs remained constant during therapy (Table 2; Fig. 1). In contrast to Cr, a highly significant decrease in CSTC was seen from week 2 onward with a subsequent increase in CSTC-based eGFR (Table 2; Fig. 2). An extend evaluation of patients with paired values until 24 weeks after therapy (*n* = 42) indicated that the decrease in CSTC was consistent through therapy (*P *< 0.0001; Fig. S2, see online supplementary material), whereas no late effect was observed for Cr (*P *= 0.1099; Fig. S2, see online supplementary material). For BTP, a significant decrease in BTP was noticed at week 2 and 4, compared to baseline, which normalized by week 8. Vice versa, the same changes were noted for the BTP-based GFR (Table 2; Fig. 3). Lastly, the GFR estimations of combined serum parameters showed a significant change from week 2 onward for all formulas in which CSTC is incorporated (Table 2; Fig. 4).

**Table 2:** Variation in biochemical measurements and eGFR during TKI treatment.

Equation	Baseline Median (IQR)	Week 2 Median (IQR)	Week 4 Median (IQR)	Week 8 Median (IQR)	*P*
1. Serum analytes					
Cr	1.08 (0.85, 1.47)	1.16 (0.89, 1.43)	1.17 (0.86, 1.38)	1.16 (0.87, 1.47)	0.7753
CSTC	1.07 (0.87, 1.42)	0.92 (0.75, 1.17)	0.94 (0.74, 1.16)	0.92 (0.81, 1.15)	<0.0001
BTP	0.66 (0.53, 0.90)	0.66 (0.46, 0.81)	0.66 (0.48, 0.82)	0.66 (0.46, 0.78)	0.0100
2. CKD-EPI eGFR					
Cr	64 (47, 79)	61 (49, 73)	65 (47, 84)	61 (48, 76)	0.7457
CSTC	69 (47, 92)	84 (62, 105)	85 (61, 106)	86 (65, 100)	<0.0001
Cr + CSTC	69 (48, 85)	74 (58, 92)	73 (55, 92)	72 (58, 89)	<0.0001
BTP	61 (49, 72)	61 (52, 79)	64 (53, 74)	63 (56, 76)	0.0089
ΔGFR Cr—CSTC	−4 (−22, 11)	−18 (−36, −4)	−19 (−33, −8)	−18 (−33, −4)	<0.0001
ΔGFR Cr—BTP	−2 (−13, 12)	−7 (−21, 11)	−5 (−18, 6)	−3 (−17, 8)	0.1138
ΔGFR CSTC—BTP	7 (−6, 21)	15 (−3, 26)	13 (−1, 35)	16 (−3, 30)	0.0196
3. FAS eGFR					
Cr	60 (46, 74)	59 (46, 75)	60 (47, 74)	59 (48, 72)	0.7937
CSTC	62 (46, 82)	73 (57, 93)	76 (57, 96)	74 (58, 86)	<0.0001
BTP	73 (50, 90)	76 (59, 102)	75 (57, 106)	76 (58, 106)	0.0070
Cr + CSTC	61 (47, 76)	65 (61, 81)	66 (51, 81)	64 (52, 80)	<0.0001
Cr + BTP	66 (50, 78)	65 (54, 81)	67 (52, 82)	66 (54, 81)	0.0302
CSTC + BTP	66 (51, 75)	75 (55, 92)	75 (54, 95)	73 (60, 94)	<0.0001
Cr + CSTC + BTP	64 (49, 75)	66 (50, 79)	69 (51, 82)	65 (52, 80)	<0.0001
ΔGFR Cr—CSTC	−2 (−14, 10)	−15 (−28, −1)	−15 (−26, −3)	−11 (−27, −3)	<0.0001
ΔGFR Cr—BTP	−8 (−31, −1)	−17 (−41, −5)	−16 (−43, −3)	−16 (−40, −2)	0.0379
ΔGFR CSTC—BTP	−11 (−26, 4)	−9 (−28, 11)	−2 (−31, 12)	−7 (−30, 10)	0.5540
4. EKFC eGFR					
Cr	63 (46, 74)	59 (46, 73)	61 (46, 77)	60 (47, 73)	0.7453
CSTC	70 (49, 86)	79 (62, 91)	80 (62, 92)	81 (65, 91)	<0.0001
ΔGFR Cr—CSTC	−7 (−19, 6)	−17 (−29, −4)	−15 (−27, −8)	−16 (−25, −7)	<0.0001

eGFR are given as mL/min/1.73m². Cr-based eGFR was estimated using the CKD-EPI 2009 equation.

BTP, β-trace protein; CKD-EPI, Chronic Kidney Disease Epidemiology Collaboration; Cr, creatinine; CSTC, cystatin C; eGFR, estimated glomerular filtration rate; EKFC, European Kidney Function Consortium; FAS, Full Age Spectrum; IQR, interquartile range; TKI, tyrosine kinase inhibitor.

**Figure 5:**
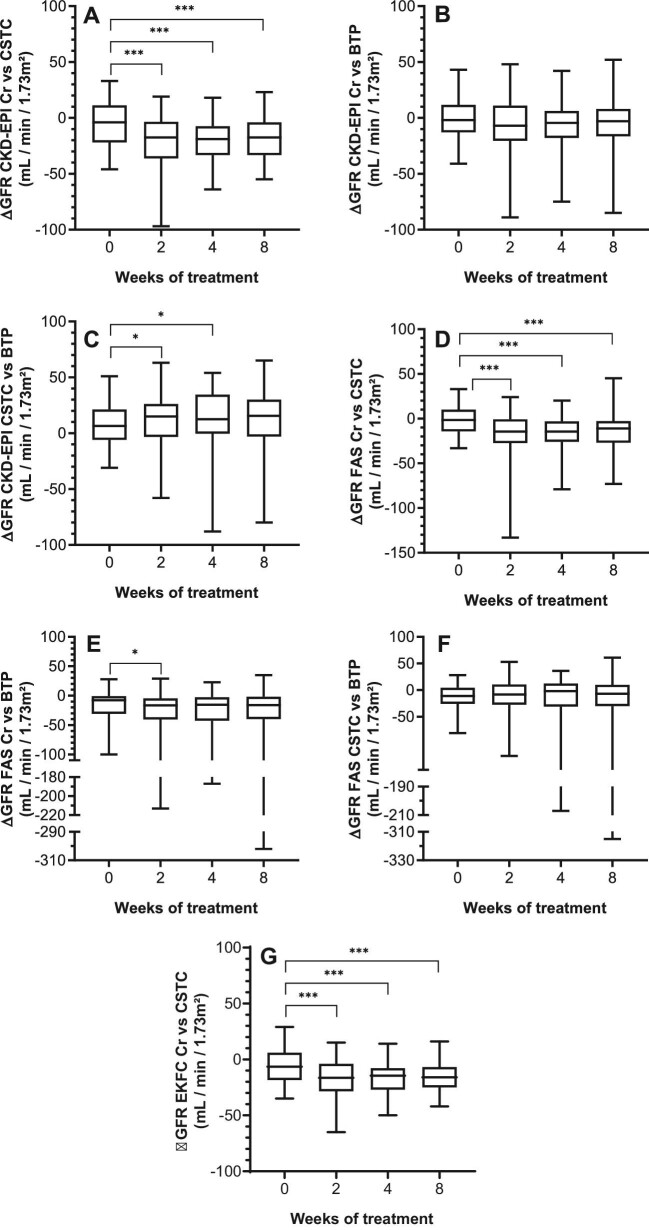
Intrapatient comparison of ΔGFR during TKI treatment. Box plots are given for the different parameters over time. X-axis depicts time of measurement. Comparisons are illustrated for: **A** ΔGFR CKD-EPI Cr vs CSTC (*P *< 0.0001); **B** ΔGFR CKD-EPI Cr vs BTP (*P *= 0.0123); **C** ΔGFR CKD-EPI CSTC vs BTP (*P *= 0.1123); **D** ΔGFR FAS Cr vs CSTC (*P *< 0.0001); **E** ΔGFR FAS Cr vs BTP (*P *= 0.0123); **F** ΔGFR FAS CSTC vs BTP (*P *= 0.1123); and **G** EKFC Cr vs CSTC (*P *< 0.0001). ΔGFR was calculated by subtracting the CSTC-based eGFR form the Cr-based eGFR, the BTP-based eGFR form the Cr-based eGFR, and the BTP-based eGFR form the CSTC-based eGFR; respectively. Intergroup significant changes are indicated in the figure (*P < 0.05; ***P < 0.001). Cr-based eGFR was estimated using the CKD-EPI 2009 equation. ΔGFR, GFR gap; BTP, β-trace protein; CKD-EPI, Chronic Kidney Disease Epidemiology Collaboration; Cr, creatinine; CSTC, cystatin C; eGFR, estimated glomerular filtration rate; EKFC, European Kidney Function Consortium; FAS, Full Age Spectrum; TKI, tyrosine kinase inhibitor.

Due to the observed changes in GFR, we determined whether the differences between GFR equations (ΔGFR), based on single biochemical analyte, were significant. All differences per time point are given in Table 2. Interestingly, all ΔGFR concerning CSTC-related eGFR were significantly different during treatment, compared to baseline. Interestingly, no significant difference was observed for ΔGFR CSTC—BTP when using the FAS equation (Table 2; Fig. 5).

Next, significant changes for urinary biomarkers were noticed. A 50% increase in median UPCR from baseline (108.3 mg/g Cr) was seen at week 2 (169.6 mg/g Cr), at week 4 (167.2 mg/g Cr), and at week 8 (157.6 mg/g Cr, *P *< 0.0001; Fig. S3A, see online supplementary material). Similarly, a threefold increase in median UACR was observed between baseline (17.5 mg/g Cr) and week 2 (49.9 mg/g Cr), reducing to a twofold increase at week 4 (36.5 mg/g Cr) and week 8 (32.0 mg/g Cr; *P *< 0.0001; Fig. S3B, see online supplementary material). The number of patients observed with proteinuria and albuminuria during the study are indicated in Table S2 (see online supplementary material). Median urine IgG significantly increased from baseline (4.0 mg/L) to week 2 (8.7 mg/L) and week 4 (6.0 mg/L), but stabilized at week 8 (5.3 mg/L; *P *< 0.0001; Fig. S3C, see online supplementary material). Lastly, no tubular kidney damage was observed with median α-1-microglobulin remaining stable throughout treatment (12.0 mg/L versus 12.4 mg/L versus 13.9 mg/L versus 10.6 mg/L; *P *= 0.1458; Fig. S3D, see online supplementary material).

In the subcohort analysis, the differences in serum parameters and associated eGFRs were evaluated in a cohort of RCC patients (*n* = 19) receiving TKIs and ICIs (or *vice versa*) and compared between treatment regimens. Cr and Cr-based eGFRs remained stable during treatment with a TKI or with an ICI (Fig. S4, see online supplementary material). For CSTC, the significant decrease in CSTC concentrations, and consequent increases in GFR estimations were also noticed in the TKI subcohort. However, no significant changes were observed in the ICI cohort (Fig. S5, see online supplementary material). The same holds true for BTP, where (only borderline) significant changes were observed in the TKI cohort, but not in the ICI cohort (Fig. S6, see online supplementary material). The treatment-related changes in urinary analytes in the TKI cohort, measured via UPCR, UACR, and IgG; were not present in the ICI cohort (Fig. S7, see online supplementary material).

Lastly, an ICI-related decrease was noticed in systolic (134 mm Hg versus 129 mm Hg versus 126 mm Hg versus 125 mm Hg; *P* = 0.0098) and diastolic blood pressure (79 mm Hg versus 74 mm Hg versus 73 mm Hg versus 73 mm Hg; *P* = 0.0858) was seen at baseline, week 2, 4, and 8, respectively.

## DISCUSSION

There have been changes in the equations to estimate GFR, as well as in the guidelines for use of these GFR estimations. As it has been reported that the eGFR may be influenced by tumor-related factors, the objective of this research paper was to evaluate several currently implemented GFR equations as kidney function monitoring tools in oncology patients treated with TKIs.

Firstly, we noticed that the systolic and diastolic blood pressure in actively treated patients increased during their treatment. This finding confirms the findings observed in the large phase III trials studying the use of various TKIs in RCC patients. In these studies, occurrence of hypertension is reported in 30% to 46% of patients receiving a TKI [6–8]. As it is widely recognized that an increase in blood pressure contributes to extensive kidney damage, the therapy-related increase in blood pressure is an initial proof of a TKI-related side effect due to therapy-related processes occurring in the kidneys [30]. In addition, Meyrier [31] stated that kidney arterial hypertension results in nephrosclerosis, which is clinically associated with proteinuria [31]. In our study, we have also noticed an increase in proteinuria, namely a 50% increase in UPCR, compared to baseline, throughout treatment, with 57% of patients having mild to severe proteinuria. This finding of TKI-related proteinuria is in line with recent reports [32, 33], including a meta-analysis from Xiong *et al*. [33] highlighting the risk of renal impairment by the administration of TKIs [33].

A review by Kandula and Agarwal [34] describes that the TKI-induced kidney damage is caused by renal VEGF inhibition, which results in an inhibited nephrin production and interruption of the glomerular filtration barrier [34]. Indeed, a more extensive evaluation indicated that the proteinuria, observed in our study, was the result of extensive glomerular damage, occurring as clinically significant albuminuria from week 2 onward (threefold increase versus baseline) with 59% of patients having mild to severe albuminuria. Surprisingly, the degree of albuminuria was more severe in male patients compared to female patients. In addition, these patients presented with increased urinary IgG. As IgG has twice the molecular weight of albumin (±150 kDa), the occurrence of TKI-related IgG-uria indicates the severe onset of potential glomerular disease [35, 36]. Correct and close monitoring of patients’ kidney function during therapy is therefore essential, especially as an elevated (systolic) blood pressure is known to contribute to a decline in kidney function [37, 38].

Determination of the eGFR is the best available indicator of the kidney function [39]. In order to estimate the GFR, a variety in formulas exist, depending on the measured biochemical parameter, such as Cr, CSTC, or BTP [14, 24, 25]. All patients in our study showed comparable baseline eGFR according to the different formulas used, except for the FAS BTP equation, which tended to overestimate the eGFR. During TKI treatment, no changes were observed in the CKD-EPI, FAS, or EKFC Cr-related eGFR. This finding confirms the initial result reported by our group [40]. This observation is, however, contradictory to literature as Ning *et al*. [32] reported an eGFR decrease in mRCC patients treated with targeted therapy. This controversy is even larger in case of CSTC and BTP as Ning *et al*. [32] reported a CSTC increase during therapy with consequent decrease in GFR [32].

In our study, applying BTP and CSTC as analyte resulted in significant changes during therapy. BTP and respective BTP-based eGFRs were shown to be significantly decreased and increased after 2 and 4 weeks of therapy, before stabilizing after 8 weeks. Using the FAS BTP equation, we did, however, observe several outliers, ranging to 441 mL/min/1.73 m². Of the six patients who had an FAS BTP-based eGFR over 200 mL/min/1.73 m², four were male, but more importantly five patients were 50 years old or younger (45% of patients below the age of 51 years old who were included in the BTP analysis). This indicates that the FAS BTP equation is greatly biased by age and thus overestimates the eGFR in younger patients, as was also reported by Pottel *et al*. [17]. The FAS BTP equation should therefore be used cautiously in younger patients who are being treated with a TKI.

For CSTC, we have noticed a decrease in CSTC that remained consistent from week 2 of therapy onward, including in patients who remained on therapy for at least 24 weeks (*n* = 41). Consequently, the CSTC-based eGFR, determined via the CKD-EPI, FAS, and EKFC equations, increased during therapy, which would falsely indicate that the patients’ kidney function is improving after TKI administration. The latter is particularly true as the calculated difference in GFR (ΔGFR), according to different formulas used, was significantly different for each ΔGFR involving the CSTC-based eGFR, except for the ΔGFR FAS CSTC—BTP due to the above-mentioned effect of age on the FAS BTP eGFR. Moreover, using combination formulas, including two or three of the serum analytes, resulted in a likewise apparent increase in eGFR for each formula in which CSTC was incorporated. Given the evidence of TKI-associated kidney damage [34], the decrease in BTP and CSTC must be related to tumoral changes following TKI administration. The question remains: which biological processes result in the apparent decrease in BTP and CSTC?

To our knowledge, the causal relationship between TKI administration and variation in BTP has not been studied in-depth. Omori *et al*. [41] indicated that an increased lipocalin-type prostaglandin D synthase expression is linked to malignant properties of tumor endothelial cells and associated tumoral neo-angiogenesis [41]. It can therefore be hypothesized that anti-VEGF cancer therapy results in a decrease in BTP concentrations. More in-depth evaluations on the effect of TKIs on BTP are warranted.

Similarly, the relationship between TKI administration and CSTC needs further assessment. CSTC is a member of the type 2 family of cysteine proteinase inhibitors, playing a significant role in cell regulation, cell proliferation, and apoptosis by regulating the breakdown of extracellular and intracellular proteins. As such, cysteine proteinase inhibitors ensure the balance between free cysteine proteinases and their complexed form. Due to their regulation against harmful cysteine proteinase activities, cysteine proteinase inhibitors have been linked to a number of pathologies [42, 43].

With regard to tumor development and angiogenesis, Dreilich *et al*. [44] indicated that baseline CSTC is correlated to VEGF in esophageal carcinoma [44]. *In vitro* research from Li *et al.* [45] demonstrated that VEGF-A blockage promotes CSTC expression, thus indicating a CSTC increase during TKI treatment, due to targeting of the VEGF axis [45]. However, we have noticed a highly significant decrease in CSTC throughout therapy, which can probably be explained by the cathepsin-inhibitory properties of CSTC. A review by Breznik *et al*. [46] highlighted that CSTC is the most potent inhibitor of cysteine peptidases [46]. Moreover, *in vitro* experiments demonstrated that tumor-derived cathepsin D reduces CSTC activity in the tumor microenvironment through cathepsin D-mediated complexation and proteolysis [47]. This was also highlighted by our group, as cathepsin D addition to serum resulted in a total CSTC depletion from serum [40]. Future in-depth evaluations are warranted, although it is clear that CSTC-based GFR estimation is not reliable nor robust for kidney monitoring of TKI-treated oncology patients.

In a final part, we assessed whether the decrease in CSTC, observed in patients after TKI administration, is also noticed for patients receiving ICI as prior or next line of therapy (*n* = 19). Conversely to the CSTC decrease during TKI therapy, a non-significant slight increase has been observed in CSTC following ICI administration. This is partly in line with the data Ning *et al.* [32] who found that PD-1 inhibitor administration did not attribute to a difference in GFR estimation [32]. Our finding indicates that the difference in mechanism of action of both TKIs and ICIs harbor a different effect on serum CSTC. The observed exacerbation in ICI-related proteinuria was not seen in our patient population [32].

A first limitation of our study is the absence of any direct measurement of GFR. Therefore, we cannot directly confirm the increase in CSTC-based eGFR. Nevertheless, no ideal method for measured GFR exists as substantial variation is reported for measured GFR results across studies and which questioned the scientific reliability of the alternative measured GFR methods as the gold standard to evaluate kidney function [48]. A second potential limitation is that CSTC was measured using a PENIA. Conflicting reports exist on the analytical variability between the PENIA and the particle-enhanced turbidimetric immunoassay for CSTC [49–52]. It is, however, unlikely that these possible variations result in the difference for CSTC observed in our study. Moreover, as our CSTC test is calibrated against the certified ERM®-DA471/IFCC standard, we are confident that the PENIA measurement can be considered reliable. Another limitation is that we only used BTP as analyte to estimate the BTP-based GFR. Inker *et al*. [25] stated that the formula including both BTP and β2-microglobulin shows similar accuracy to the CKD-EPI Cr or CSTC equation [25]. As we do not have any β2-microglobulin available in our patient population, we were unable to apply this equation.

## Conclusion

TKI administration is associated with treatment-related kidney damage. Although larger clinical trials do not emphasize the importance of kidney function follow-up, our data along with literature evince the cruciality for a close patient monitoring during TKI administration for potentially severe nephropathy. It remains, however, debatable which GFR estimation should be implemented as the ‘gold standard’. Despite the fact that the Cr-based GFR estimation remains constant during TKI treatment, the CSTC-based GFR significantly increased during therapy with TKIs, raising questions on the accuracy of these estimates in oncology patients treated with TKIs. In line with the suggestions of Levey *et al*. [53], we propose for an extensive evaluation into the mechanisms that play a pivotal role in the observed CSTC decrease, as well as the continued optimization of GFR estimations.

## Supplementary Material

sfae006_Supplemental_File

## Data Availability

All data generated and analysed during this study cannot be shared publicly due to the European General Data Protection Regulation (EU GDPR). The data will be shared on reasonable request to the corresponding author, following approval of the data protection officer of the University Hospital Ghent.
