# Supplementary material for: Comparison of different estimated glomerular filtration rates for monitoring of kidney function in oncology patients
Source: Clin Kidney J. 2024 Jan 12;17(1):sfae006. doi: 10.1093/ckj/sfae006 (PMC10823486; doi:10.1093/ckj/sfae006)
Supplement: sfae006_Supplemental_File [file sfae006_supplemental_file.docx]

**Original article – Supplementary files**


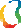

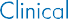

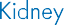

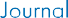


**Supplementary Tables:**

| ***Table S1: Formulas for eGFR used in this paper*** | | | | |
| --- | --- | --- | --- | --- |
| ***Equation*** | ***Age*** | ***Sex*** | ***Condition*** | ***eGFR Formula*** |
| 1. *CKD-EPI* |  |  |  |  |
| Cr ^2009^ (1) | ≥ 18 | F | S_Cr_ ≤ 0.70 | 144 x [S_Cr_ / 0.70]^-0.329^ x 0.9929^Age^ |
|  | ≥ 18 | F | S_Cr_ > 0.70 | 144 x [S_Cr_ / 0.70]^-1.209^ x 0.9929^Age^ |
|  | ≥ 18 | M | S_Cr_ ≤ 0.90 | 141 x [S_Cr_ / 0.90]^-0.411^ x 0.9929^Age^ |
|  | ≥ 18 | M | S_Cr_ > 0.90 | 141 x [S_Cr_ / 0.90]^-1.209^ x 0.9929^Age^ |
| Cr ^2021^ (2) | ≥ 18 | F | S_Cr_ ≤ 0.70 | 143 x [S_Cr_ / 0.70]^-0.241^ x 0.9938^Age^ |
|  | ≥ 18 | F | S_Cr_ > 0.70 | 143 x [S_Cr_ / 0.70]^-1.200^ x 0.9938^Age^ |
|  | ≥ 18 | M | S_Cr_ ≤ 0.90 | 142 x [S_Cr_ / 0.90]^-0.302^ x 0.9938^Age^ |
|  | ≥ 18 | M | S_Cr_ > 0.90 | 142 x [S_Cr_ / 0.90]^-1.200^ x 0.9938^Age^ |
| CSTC (3) | ≥ 18 | F | S_CSTC_ ≤ 0.80 | 133 x (S_CSTC_ / 0.80)^-0.499^ x 0.9962^Age^ x 0.932 |
|  | ≥ 18 | F | S_CSTC_ > 0.80 | 133 x (S_CSTC_ / 0.80)^-1.328^ x 0.9962^Age^ x 0.932 |
|  | ≥ 18 | M | S_CSTC_ ≤ 0.80 | 133 x (S_CSTC_ / 0.80)^-0.499^ x 0.9962^Age^ |
|  | ≥ 18 | M | S_CSTC_ > 0.80 | 133 x (S_CSTC_ / 0.80)^-1.328^ x 0.9962^Age^ |
| Cr ^2009^ + CSTC (3) | ≥ 18 | F | S_Cr_ ≤ 0.70 and Scstc ≤ 0.80 | 130 x (S_Cr_ / 0.70)^-0.248^ x (S_CSTC_ / 0.80)^-0.375^ x 0.9952^Age^ |
|  | ≥ 18 | F | S_Cr_ ≤ 0.70 and Scstc > 0.80 | 130 x (S_Cr_ / 0.70)^-0.248^ x (S_CSTC_ / 0.80)^-0.711^ x 0.9952^Age^ |
|  | ≥ 18 | F | S_Cr_ > 0.70 and Scstc ≤ 0.80 | 130 x (S_Cr_ / 0.70)^-0.601^ x (S_CSTC_ / 0.80)^-0.375^ x 0.9952^Age^ |
|  | ≥ 18 | F | S_Cr_ > 0.70 and Scstc > 0.80 | 130 x (S_Cr_ / 0.70)^-0.601^ x (S_CSTC_ / 0.80)^-0.711^ x 0.9952^Age^ |
|  | ≥ 18 | M | S_Cr_ ≤ 0.90 and Scstc ≤ 0.80 | 135 x (S_Cr_ / 0.90)^-0.207^ x (S_CSTC_ / 0.80)^-0.375^ x 0.9952^Age^ |
|  | ≥ 18 | M | S_Cr_ ≤ 0.90 and Scstc > 0.80 | 135 x (S_Cr_ / 0.90)^-0.207^ x (S_CSTC_ / 0.80)^-0.711^ x 0.9952^Age^ |
|  | ≥ 18 | M | S_Cr_ > 0.90 and Scstc ≤ 0.80 | 135 x (S_Cr_ / 0.90)^-0.601^ x (S_CSTC_ / 0.80)^-0.375^ x 0.9952^Age^ |
|  | ≥ 18 | M | S_Cr_ > 0.90 and Scstc > 0.80 | 135 x (S_Cr_ / 0.90)^-0.601^ x (S_CSTC_ / 0.80)^-0.711^ x 0.9952^Age^ |
| Cr ^2021^ + CSTC (2) | ≥ 18 | F | S_Cr_ ≤ 0.70 and Scstc ≤ 0.80 | 130 x (S_Cr_ / 0.70)^-0.219^ x (S_CSTC_ / 0.80)^-0.323^ x 0.9961^Age^ |
|  | ≥ 18 | F | S_Cr_ ≤ 0.70 and Scstc > 0.80 | 130 x (S_Cr_ / 0.70)^-0.219^ x (S_CSTC_ / 0.80)^-0.778^ x 0.9961^Age^ |
|  | ≥ 18 | F | S_Cr_ > 0.70 and Scstc ≤ 0.80 | 130 x (S_Cr_ / 0.70)^-0.544^ x (S_CSTC_ / 0.80)^-0.323^ x 0.9961^Age^ |
|  | ≥ 18 | F | S_Cr_ > 0.70 and Scstc > 0.80 | 130 x (S_Cr_ / 0.70)^-0.544^ x (S_CSTC_ / 0.80)^-0.778^ x 0.9961^Age^ |
|  | ≥ 18 | M | S_Cr_ ≤ 0.90 and Scstc ≤ 0.80 | 135 x (S_Cr_ / 0.90)^-0.144^ x (S_CSTC_ / 0.80)^-0.323^ x 0.9961^Age^ |
|  | ≥ 18 | M | S_Cr_ ≤ 0.90 and Scstc > 0.80 | 135 x (S_Cr_ / 0.90)^-0.144^ x (S_CSTC_ / 0.80)^-0.778^ x 0.9961^Age^ |
|  | ≥ 18 | M | S_Cr_ > 0.90 and Scstc ≤ 0.80 | 135 x (S_Cr_ / 0.90)^-0.544^ x (S_CSTC_ / 0.80)^-0.323^ x 0.9961^Age^ |
|  | ≥ 18 | M | S_Cr_ > 0.90 and Scstc > 0.80 | 135 x (S_Cr_ / 0.90)^-0.544^ x (S_CSTC_ / 0.80)^-0.778^ x 0.9961^Age^ |
| BTP (4) | ≥ 18 | F |  | 55 x S_BTP_^-0.695^ x 0.998^Age^ x 0.899 |
|  | ≥ 18 | M |  | 55 x S_BTP_^-0.695^ x 0.998^Age^ |
| 1. *FAS* |  |  |  |  |
| Cr (5, 6) | ≤ 40 | F |  | 107.3 / [S_Cr_ / 0.70] |
|  | > 40 | F |  | 107.3 / [S_Cr_ / 0.70] x 0.988^(Age-40)^ |
|  | ≤ 40 | M |  | 107.3 / [S_Cr_ / 0.90] |
|  | > 40 | M |  | 107.3 / [S_Cr_ / 0.90] x 0.988^(Age-40)^ |
| CSTC (5, 6) | ≤ 40 | - |  | 107.3 / [S_CSTC_ / 0.82] |
|  | 40-75 | - |  | 107.3 / [S_CSTC_ / 0.82] x 0.988^(Age-40)^ |
|  | > 75 | - |  | 107.3 / [S_CSTC_ / 0.95] x 0.988^(Age-40)^ |
| BTP (6) | ≤ 40 | - |  | 107.3 / [S_BTP_ / 0.60] |
|  | > 40 | - |  | 107.3 / [S_BTP_ / 0.60] x 0.988^(Age-40)^ |
| Cr + CSTC (5, 6) | ≤ 40 | F |  | 107.3 / [0.5 x S_Cr_ / 0.70 + 0.5 x S_CSTC_ / 0.82] |
|  | 40-75 | F |  | 107.3 / [0.5 x S_Cr_ / 0.70 + 0.5 x S_CSTC_ / 0.82] x 0.988^(Age-40)^ |
|  | > 75 | F |  | 107.3 / [0.5 x S_Cr_ / 0.70 + 0.5 x S_CSTC_ / 0.95] x 0.988^(Age-40)^ |
|  | ≤ 40 | M |  | 107.3 / [0.5 x S_Cr_ / 0.90 + 0.5 x S_CSTC_ / 0.82] |
|  | 40-75 | M |  | 107.3 / [0.5 x S_Cr_ / 0.90 + 0.5 x S_CSTC_ / 0.82] x 0.988^(Age-40)^ |
|  | > 75 | M |  | 107.3 / [0.5 x S_Cr_ / 0.90 + 0.5 x S_CSTC_ / 0.95] x 0.988^(Age-40)^ |
| Cr + BTP (5, 6) | ≤ 40 | F |  | 107.3 / [0.5 x S_Cr_ / 0.70 + 0.5 x S_BTP_ / 0.60] |
|  | > 40 | F |  | 107.3 / [0.5 x S_Cr_ / 0.70 + 0.5 x S_BTP_ / 0.60] x 0.988^(Age-40)^ |
|  | ≤ 40 | M |  | 107.3 / [0.5 x S_Cr_ / 0.90 + 0.5 x S_BTP_ / 0.60] |
|  | > 40 | M |  | 107.3 / [0.5 x S_Cr_ / 0.90 + 0.5 x S_BTP_ / 0.60] x 0.988^(Age-40)^ |
| CSTC + BTP (5, 6) | ≤ 40 | - |  | 107.3 / [0.5 x Scstc/0.82 + 0.5 x S_BTP_ / 0.60] |
|  | 40-75 | - |  | 107.3 / [0.5 x Scstc/0.82 + 0.5 x S_BTP_ / 0.60] x 0.988^(Age-40)^ |
|  | > 75 | - |  | 107.3 / [0.5 x Scstc/0.95 + 0.5 x S_BTP_ / 0.60] x 0.988^(Age-40)^ |
| Cr + CSTC + BTP (5, 6) | ≤ 40 | F |  | 107.3 / [0.39 x S_Cr_ / 0.70 + 0.38 x S_CSTC_ / 0.82 + 0.23 x S_BTP_ / 0.60] |
|  | 40-75 | F |  | 107.3 / [0.39 x S_Cr_ / 0.70 + 0.38 x S_CSTC_ / 0.82 + 0.23 x S_BTP_ / 0.60] x 0.988^(Age-40)^ |
|  | > 75 | F |  | 107.3 / [0.39 x S_Cr_ / 0.70 + 0.38 x S_CSTC_ / 0.95 + 0.23 x S_BTP_ / 0.60] x 0.988^(Age-40)^ |
|  | ≤ 40 | M |  | 107.3 / [0.39 x S_Cr_ / 0.90 + 0.38 x S_CSTC_ / 0.82 + 0.23 x S_BTP_ / 0.60] |
|  | 40-75 | M |  | 107.3 / [0.39 x S_Cr_ / 0.90 + 0.38 x S_CSTC_ / 0.82 + 0.23 x S_BTP_ / 0.60] x 0.988^(Age-40)^ |
|  | > 75 | M |  | 107.3 / [0.39 x S_Cr_ / 0.90 + 0.38 x S_CSTC_ / 0.95 + 0.23 x S_BTP_ / 0.60] x 0.988^(Age-40)^ |
| 1. *EKFC* |  |  |  |  |
| Cr (7, 8) | ≤ 40 | F | S_Cr_ / 0.70 < 1.0 | 107.3 x [S_Cr_ / 0.70]^-0.322^ |
|  | ≤ 40 | F | S_Cr_ / 0.70 ≥ 1.0 | 107.3 x [S_Cr_ / 0.70]^-1.132^ |
|  | ≤ 40 | M | S_Cr_ / 0.90 < 1.0 | 107.3 x [S_Cr_ / 0.90]^-0.322^ |
|  | ≤ 40 | M | S_Cr_ / 0.90 ≥ 1.0 | 107.3 x [S_Cr_ / 0.90]^-1.132^ |
|  | > 40 | F | S_Cr_ / 0.70 < 1.0 | 107.3 x [S_Cr_ / 0.70]^-0.322^ x 0.990^(Age-40)^ |
|  | > 40 | F | S_Cr_ / 0.70 ≥ 1.0 | 107.3 x [S_Cr_ / 0.70]^-1.132^ x 0.990^(Age-40)^ |
|  | > 40 | M | S_Cr_ / 0.90 < 1.0 | 107.3 x [S_Cr_ / 0.90]^-0.322^ x 0.990^(Age-40)^ |
|  | > 40 | M | S_Cr_ / 0.90 ≥ 1.0 | 107.3 x [S_Cr_ / 0.90]^-1.132^ x 0.990^(Age-40)^ |
| CSTC (8) | ≤ 40 | - | S_CSTC_ / 0.83 < 1.0 | 107.3 x [S_CSTC_ / 0.83]^-0.322^ |
|  | ≤ 40 | - | S_CSTC_ / 0.83 ≥ 1.0 | 107.3 x [S_CSTC_ / 0.83]^-1.132^ |
|  | 40-50 | - | S_CSTC_ / 0.83 < 1.0 | 107.3 x [S_CSTC_ / 0.83]^-0.322^ x 0.988^(Age-40)^ |
|  | 40-50 | - | S_CSTC_ / 0.83 ≥ 1.0 | 107.3 x [S_CSTC_ / 0.83]^-1.132^ x 0.988^(Age-40)^ |
|  | > 50 | - | S_CSTC_ / [0.83+0.005 x (Age-50)] < 1.0 | 107.3 x [S_CSTC_ / (0.83+0.005 x [Age-50])]^-0.322^ x 0.988^(Age-40)^ |
|  | > 50 | - | S_CSTC_ / [0.83+0.005 x (Age-50)] ≥ 1.0 | 107.3 x [S_CSTC_ / (0.83+0.005 x [Age-50])]^-1.132^ x 0.988^(Age-40)^ |
| BTP, β-trace protein; CKD-EPI, Chronic Kidney Disease Epidemiology Collaboration; Cr, creatinine; CSTC, cystatin C; EKFC, European Kidney Function Consortium; F, female; FAS, Full Age Spectrum; M, male; S, serum. | | | | |

| ***Table S2: Severity of proteinuria and albuminuria in patients receiving TKI treatment*** | | | | | | |
| --- | --- | --- | --- | --- | --- | --- |
| **Time** | **N** | **Number of patients with value above upper limit** | | | | **Highest value** |
| *Proteinuria (UPCR)* | | | | | | |
| *All patients* |  | *>150 mg/g Cr (mild - severe) ** | | *> 500 mg/g Cr (severe) ** | |  |
| Baseline | 103 | 37 | (36%) | 15 | (15%) | 2759.3 mg/g Cr |
| Week 2 | 99 | 56 | (57%) | 19 | (19%) | 6157.5 mg/g Cr |
| Week 4 | 98 | 54 | (55%) | 17 | (17%) | 5434.7 mg/g Cr |
| Week 8 | 77 | 41 | (53%) | 11 | (14%) | 5779.4 mg/g Cr |
| Week 12 | 67 | 33 | (49%) | 12 | (18%) | 8113.6 mg/g Cr |
| Week 24 | 50 | 23 | (46%) | 7 | (14%) | 4132.4 mg/g Cr |
| *Albuminuria (UACR)* | | | | | | |
| *All patients* |  | *>30 mg/g Cr (mild - severe) ** | | *>300 mg/g Cr (severe) ** | |  |
| Baseline | 102 | 36 | (35%) | 10 | (10%) | 2215.7 mg/g Cr |
| Week 2 | 98 | 58 | (59%) | 16 | (16%) | 5116.6 mg/g Cr |
| Week 4 | 96 | 50 | (52%) | 14 | (15%) | 4338.9 mg/g Cr |
| Week 8 | 77 | 41 | (53%) | 10 | (13%) | 4626.9 mg/g Cr |
| Week 12 | 66 | 39 | (59%) | 10 | (15%) | 6210.3 mg/g Cr |
| Week 24 | 49 | 22 | (45%) | 5 | (10%) | 3181.8 mg/g Cr |
| *Female patients* |  | *> 25 mg/g Cr (mild to severe) ^§^* | | | |  |
| Baseline | 102 | 12 | | (32%) | | 520.0 mg/g Cr |
| Week 2 | 98 | 21 | | (57%) | | 423.5 mg/g Cr |
| Week 4 | 96 | 21 | | (58%) | | 374.7 mg/g Cr |
| Week 8 | 77 | 19 | | (58%) | | 268.0 mg/g Cr |
| Week 12 | 66 | 18 | | (60%) | | 6210.3 mg/g Cr |
| Week 24 | 49 | 13 | | (59%) | | 342.1 mg/g Cr |
| *Male patients* |  | *> 17 mg/g Cr (mild to severe) ^§^* | | | |  |
| Baseline | 102 | 34 | | (53%) | | 2215.7 mg/g Cr |
| Week 2 | 98 | 43 | | (70%) | | 5116.6 mg/g Cr |
| Week 4 | 96 | 41 | | (68%) | | 4338.9 mg/g Cr |
| Week 8 | 77 | 32 | | (73%) | | 4626.9 mg/g Cr |
| Week 12 | 66 | 24 | | (67%) | | 2545.5 mg/g Cr |
| Week 24 | 49 | 19 | | (70%) | | 3181.8 mg/g Cr |
| * according to KDIGO 2012 (9)  § according to the gender-specific cutoff values, described by Mattix et al. (10)  Cr, creatinine; TKI, tyrosine kinase inhibitor; UACR, urine albumin-to-creatinine ratio; UPCR, urine protein-to-creatinine ratio. | | | | | | |

**Supplementary figures + figure legends:**


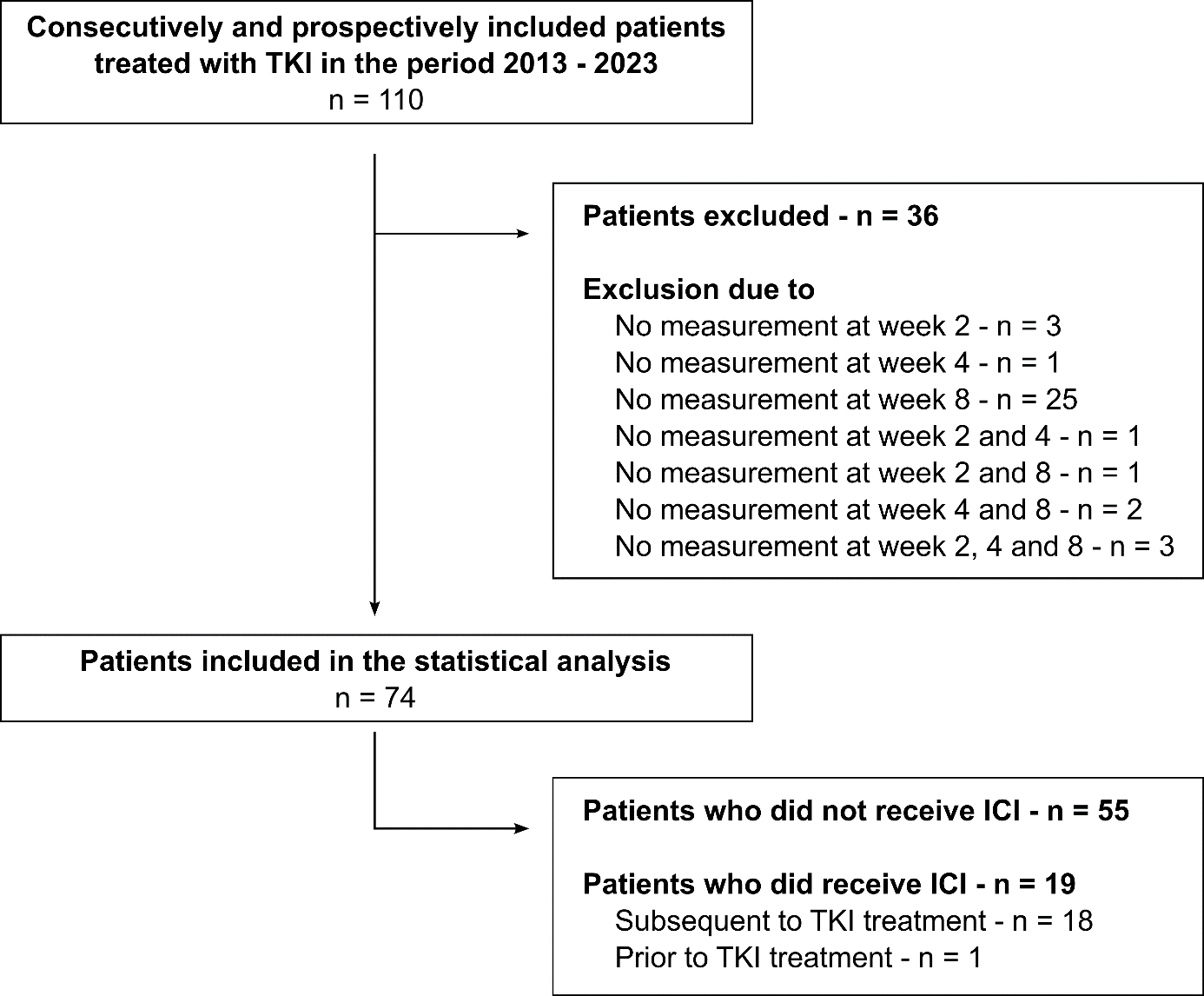


**Figure S1: STARD diagram for the study**

ICI, immune checkpoint inhibitor; TKI, tyrosine kinase inhibitor.

**Figure S2: Intrapatient comparison of Cr and CSTC concentrations until 24 weeks of TKI therapy**

Box plots are given for the different parameters over time. X-axis depicts time of measurement. Comparisons are illustrated for: A. Cr concentrations (P<0.0001), and B. CSTC concentrations (P<0.0001). Intergroup significant changes are indicated in the figure (*** P<0.001). Cr, creatinine; CSTC, cystatin C; TKI, tyrosine kinase inhibitor.

**Figure S3: Intrapatient comparison of urine analytes during TKI treatment**

Box plots are given for the different parameters over time. X-axis depicts time of measurement. Comparisons are illustrated for: A. UPCR (P<0.0001); B. UACR (P<0.0001); C. IgG (P<0.0001); and D. α-1-microglobulin (P=0.1458). Intergroup significant changes are indicated in the figure (* P<0.05; ** P<0.01; *** P<0.001). IgG, immunoglobulin G; TKI, tyrosine kinase inhibitor; UACR, urine albumin-to-creatinine ratio; UPCR, urine protein-to-creatinine ratio.

**Figure S4: Intrapatient comparison of Cr and Cr-based eGFR between TKI and ICI therapy**

Box plots are given for the different parameters over time. X-axis depicts time of measurement. Subcohort comparisons (n=19) are illustrated for: A. Cr concentrations during TKI treatment (P=0.5566); B. Cr concentrations during ICI treatment (P=0.2262); C. CKD-EPI Cr-based eGFR during TKI treatment (P=0.5199); D. CKD-EPI Cr-based eGFR during ICI treatment (P=0.2392); E. FAS Cr-based eGFR during TKI treatment (P=0.5577); F. FAS Cr-based eGFR during ICI treatment (P=0.2019), G. EFKC Cr-based eGFR during TKI treatment (P=0.5675); and H. EFKC Cr-based eGFR during ICI treatment (P=0.2256). CKD-EPI Cr-based GFR was estimated using the CKD-EPI Cr 2009 equation. Cr, creatinine; eGFR, estimated glomerular filtration rate; ICI, immune checkpoint inhibitor; TKI, tyrosine kinase inhibitor.

**Figure S5: Intrapatient comparison of CSTC and CSTC-based eGFR between TKI and ICI therapy**

Box plots are given for the different parameters over time. X-axis depicts time of measurement. Subcohort comparisons (n=19) are illustrated for: A. CSTC concentrations during TKI treatment (P=0.0066); B. CSTC concentrations during ICI treatment (P=0.7341); C. CKD-EPI CSTC-based eGFR during TKI treatment (P=0.0056); D. CKD-EPI CSTC-based eGFR during ICI treatment (P=0.7293); E. FAS CSTC-based eGFR during TKI treatment (P=0.0056); F. FAS CSTC-based eGFR during ICI treatment (P=0.6023), G. EFKC CSTC-based eGFR during TKI treatment (P=0.0022); and H. EFKC CSTC-based eGFR during ICI treatment (P=0.5440). Intergroup significant changes are indicated in the figure (* P<0.05; ** P<0.01). CSTC, cystatin C; eGFR, estimated glomerular filtration rate; ICI, immune checkpoint inhibitor; TKI, tyrosine kinase inhibitor.

**Figure S6: Intrapatient comparison of BTP and BTP-based eGFR between TKI and ICI therapy**

Box plots are given for the different parameters over time. X-axis depicts time of measurement. Subcohort comparisons (n=19) are illustrated for: A. BTP concentrations during TKI treatment (P=0.0483); B. BTP concentrations during ICI treatment (P=0.3592); C. CKD-EPI BTP-based eGFR during TKI treatment (P=0.0758); D. CKD-EPI BTP-based eGFR during ICI treatment (P=0.3087); E. FAS BTP-based eGFR during TKI treatment (P=0.0799); and F. FAS BTP-based eGFR during ICI treatment (P=0.3116). Intergroup significant changes are indicated in the figure (* P<0.05). BTP, β-trace protein; eGFR, estimated glomerular filtration rate; ICI, immune checkpoint inhibitor; TKI, tyrosine kinase inhibitor.

**Figure S7: Intrapatient comparison of UPCR, UACR and IgG between TKI and ICI therapy**

Box plots are given for the different parameters over time. X-axis depicts time of measurement. Subcohort comparisons (n=19) are illustrated for: A. UPCR during TKI treatment (P=0.0085); B. UPCR during ICI treatment (P=0.1604); C. UACR during TKI treatment (P=0.0049); D. UACR during ICI treatment (P=0.4726); E. IgG concentrations during TKI treatment (P=0.1035); F. IgG concentrations during ICI treatment (P=0.6058). Intergroup significant changes are indicated in the figure (* P<0.05; ** P<0.01; *** P<0.001). ICI, immune checkpoint inhibitor; IgG, immunoglobulin G; TKI, tyrosine kinase inhibitor; UACR, urine albumin-to-creatinine ratio; UPCR, urine protein-to-creatinine ratio.

**References**

1. Levey AS, Stevens LA, Schmid CH, et al.; A new equation to estimate glomerular filtration rate. Ann Intern Med 2009; 150(9):604-12.

2. Inker LA, Eneanya ND, Coresh J, et al.; New Creatinine- and Cystatin C-Based Equations to Estimate GFR without Race. N Engl J Med 2021; 385(19):1737-1749.

3. Inker LA, Schmid CH, Tighiouart H, et al.; Estimating glomerular filtration rate from serum creatinine and cystatin C. N Engl J Med 2012; 367(1):20-9.

4. Inker LA, Tighiouart H, Coresh J, et al.; GFR Estimation Using beta-Trace Protein and beta2-Microglobulin in CKD. Am J Kidney Dis 2016; 67(1):40-8.

5. Pottel H, Delanaye P, Schaeffner E, et al.; Estimating glomerular filtration rate for the full age spectrum from serum creatinine and cystatin C. Nephrol Dial Transplant 2017; 32(3):497-507.

6. Pottel H, Schaeffner E, Ebert N; Evaluating the diagnostic value of rescaled beta-trace protein in combination with serum creatinine and serum cystatin C in older adults. Clin Chim Acta 2018; 480:206-213.

7. Pottel H, Bjork J, Courbebaisse M, et al.; Development and Validation of a Modified Full Age Spectrum Creatinine-Based Equation to Estimate Glomerular Filtration Rate : A Cross-sectional Analysis of Pooled Data. Ann Intern Med 2021; 174(2):183-191.

8. Pottel H, Bjork J, Rule AD, et al.; Cystatin C-Based Equation to Estimate GFR without the Inclusion of Race and Sex. N Engl J Med 2023; 388(4):333-343.

9. Stevens PE, Levin A, Kidney Disease: Improving Global Outcomes Chronic Kidney Disease Guideline Development Work Group M; Evaluation and management of chronic kidney disease: synopsis of the kidney disease: improving global outcomes 2012 clinical practice guideline. Ann Intern Med 2013; 158(11):825-30.

10. Mattix HJ, Hsu CY, Shaykevich S, Curhan G; Use of the albumin/creatinine ratio to detect microalbuminuria: implications of sex and race. J Am Soc Nephrol 2002; 13(4):1034-1039.
